# ANCA Status or Clinical Phenotype — What Counts More?

**DOI:** 10.1007/s11926-021-01002-0

**Published:** 2021-04-28

**Authors:** Martin Windpessl, Erica L. Bettac, Philipp Gauckler, Jae Il Shin, Duvuru Geetha, Andreas Kronbichler

**Affiliations:** 1 Department of Internal Medicine IV, Section of Nephrology, Klinikum Wels-Grieskirchen, Grieskirchnerstrasse 42, 4600 Wels, Austria; 2 Medical Faculty, Johannes Kepler University Linz, Altenberger Strasse 69, 4040 Linz, Austria; 3 Department of Psychology, Washington State University Vancouver, 14204 NE Salmon Creek Avenue, Vancouver, WA USA; 4 Department of Internal Medicine IV (Nephrology and Hypertension), Medical University Innsbruck, Anichstrasse 35, 6020 Innsbruck, Austria; 5 Department of Pediatrics, Yonsei University College of Medicine, Seoul, 03722 Republic of Korea; 6 Division of Nephrology, Department of Medicine, Johns Hopkins University School of Medicine, Baltimore, MD USA

**Keywords:** Vasculitis, ANCA, Granulomatosis with polyangiitis, Microscopic polyangiitis, AAV

## Abstract

**Purpose of Review:**

There is ongoing debate concerning the classification of antineutrophil cytoplasmic antibody (ANCA)-associated vasculitis. That is, whether classification should be based on the serotype (proteinase 3 (PR3)- or myeloperoxidase (MPO)-ANCA) or on the clinical phenotype (granulomatosis with polyangiitis (GPA) or microscopic polyangiitis (MPA)). To add clarity, this review focused on integration of the most recent literature.

**Recent Findings:**

Large clinical trials have provided evidence that a serology-based risk assessment for relapses is more predictive than distinction based on the phenotype. Research conducted in the past decade indicated that a serology-based approach more closely resembles the genetic associations, the clinical presentation (i.e., lung involvement), biomarker biology, treatment response, and is also predicting comorbidities (such as cardiovascular death).

**Summary:**

Our review highlights that a serology-based approach could replace a phenotype-based approach to classify ANCA-associated vasculitides. In future, clinical trials and observational studies will presumably focus on this distinction and, as such, translate into a “personalized medicine.”

## Introduction

Since the groundbreaking work of van de Woude et al. [1] in the 1980s, antineutrophil cytoplasmic antibodies (ANCA) have become indispensable in the diagnostic workup of vasculitides. They are useful for classification purposes and serve as markers of disease activity as well as predictors of relapses, all with due limitations [2]. Two major ANCA types exist: directed against proteinase 3 (PR3) and myeloperoxidase (MPO). ANCA-associated vasculitides (AAV) are a group of autoimmune disorders characterized by necrotizing inflammation of small- to medium-sized blood vessels, with a propensity for lung and kidney involvement. Granulomatosis with polyangiitis (GPA) and microscopic polyangiitis (MPA) are the two major clinical syndromes. PR3-ANCA is mainly associated with GPA and MPO-ANCA with MPA. Substantial overlap exists and discordant ANCA type (e.g., MPO-positive GPA) is sometimes observed in certain populations, particularly in Asia [3, 4].

The traditional nomenclature is predominantly based on the clinical phenotype. Despite considerable overlap, clear differences between PR3-AAV and MPO-AAV regarding epidemiologic and genetic background, as well as clinical course, have recently become apparent. There is growing recognition that the ANCA status (PR3 or MPO), rather than disease type (GPA or MPA), yields more valid clinical information. Current clinical trials already account for this by stratifying enrollment according to ANCA serotype [5••]. Indeed, the last decade has seen many developments in disease pathogenesis, biomarkers, and risk stratification. Thus, in the present review, we outline recent advancements in the field and propose how diseases summarized under the umbrella of AAV might be best classified. This review will not only help to clarify current underpinnings of AAV but will also inform future directions of research.

## AAV — Nomenclature and General Aspects

The AAV share many similarities in mode of presentation, clinical course, and response to treatment. Accordingly, for a long time, GPA and MPA have been regarded as parts of a single disease spectrum and were frequently studied together in clinical trials [5•, 6, 7]. To a lesser extent, ANCA have also been found in a proportion of patients with eosinophilic GPA (EGPA), with the majority of which exhibiting MPO-ANCA. Although EGPA is presently also included in the 2012 Chapel Hill Consensus Conference (CHCC2012) nomenclature of AAV [8], it is widely considered a separate entity and was not included in major AAV trials [9]. It is therefore not addressed in this review.

While the majority of patients with AAV are positive for one ANCA subtype, a few patients have both PR3- and MPO-ANCA. A study from Korea found 10 cases of dual positivity in a cohort of 85 patients with biopsy-confirmed AAV. Kidney dysfunction was more pronounced in subjects with dual positivity, and upper airway involvement occurred more frequently in this group as compared to MPO-ANCA positive patients [10]. ANCA are also present in the serum of one-third of patients with anti-GBM disease (“double-positive” serology) [11]. A study from four European centers found that in this subgroup of patients (in total 646 cases, 568 with single-positive AAV, 41 with single-positive anti-GBM-disease, and 37 patients who were double positive), anti-MPO antibodies were more prevalent than anti-PR3 antibodies (70% vs. 27%) [12••]. Such individuals express a mixed clinical phenotype with disease severity at onset resembling pure anti-GBM disease and a propensity for relapse that is characteristic for AAV.

As proposed by the CHCC2012, vasculitides are classified according to the caliber of involved vessels. There is long-standing debate as to whether the clinical phenotype is an ideal way for classification. The disagreement among experts results from the fact that close similarities exist between GPA and MPA from a clinical point of view, and some contend categorization should follow ANCA serotype [13]. CHCC2012 thus recommended the addition of ANCA type as a prefix to the clinical phenotype (i.e., MPO-ANCA MPA, PR3-ANCA GPA, or ANCA-negative AAV) [8]. Even allowing some overlap, in single cases, it may be difficult to reconcile a positive ANCA serology with vessel size, as illustrated by a recent publication of the French Vasculitis Study Group (FVSG). In this nationwide case control study, Delaval et al. [14•] described 50 patients with AAV and temporal artery involvement, i.e., large vessel vasculitis (confirmed by biopsy in 42/50, where small-branch vasculitis and fibrinoid necrosis were distinguishing features). Despite an initial suspicion of giant cell arteritis, a diagnosis of AAV was ultimately maintained.

The prevalence of AAV is increasing, in part due to better awareness and more liberal ANCA testing, in part because of a higher survival of patients due to better therapies, but in part also due to a rise of the frequency of the disease. In spite of the bristling pace of evolution of treatment regimens, AAV still carry a poor prognosis, with a fatality rate of 28% at 5 years and survivors facing substantial morbidity resulting from the disease and its therapies [15]. The etiology of AAV is still unknown.

## ANCA Testing

ANCA are autoantibodies (predominantly of the immunoglobulin G (IgG) isotope) directed against cytoplasmic antigens expressed in the primary granules of neutrophils and lysosomes of monocytes. ANCA are regarded as pathogenic, as convincingly shown in animal models for the MPO-ANCA subtype [16]; they can activate neutrophils and monocytes, thereby mediating vascular inflammation.

ANCA remain the most valuable diagnostic serological marker in vasculitis, but — as holds true for all biomarkers — they lack absolute specificity and sensitivity and must therefore be interpreted in the context of the full clinical picture. In other words, a negative ANCA test does not rule out a diagnosis of AAV when the scenario is suggestive, especially in patients with non-renal or locally limited disease [17]. Up to a third of patients with a clinical presentation compatible with AAV are ANCA-negative by routine testing [18]. On the other hand, ANCA positivity can also occur in the absence of clinically manifest vasculitis, for instance, in cases with infections, malignancy, or secondary to certain drugs. A particular association has been reported for levamisole-altered cocaine or the prescription of drugs used in the management of hyperthyroidism (propylthiouracil, methimazole, or carbimazole) [19–21]. In these situations, ANCA directed against MPO are more frequently found than antibodies against PR3 [22, 23].

Two methods exist for ANCA detection: (1) the traditional immunofluorescence test on fixed neutrophils (IF) and (2) enzyme-linked immunosorbent assays (ELISA). In AAV, IF typically exhibits two staining patterns, scored as cytoplasmic, with proteinase 3 (PR3) as the antigen, or as perinuclear, with myeloperoxidase (MPO) specificity. A two-step diagnostic approach (IF followed by ELISA) was the conventional laboratory method. A multicenter study from the European Vasculitis Society (EUVAS) [24] compared the results of two IF methods and 8 different immunoassays. Sera from 251 incident AAV patients were compared with those of > 900 patients in whom ANCA testing had been performed but vasculitis subsequently excluded. Strikingly, most new generation ELISA outperformed IF for ANCA determination. Given the excellent performance of modern immunoassays, an international expert consensus recommended initial testing with high-quality ELISA and posited that screening with IF could be abandoned in many cases [25]. A recent meta-analysis found a superior immunoassay sensitivity and confirms that screening for PR3- or MPO-ANCA improves the diagnostic workup of AAV [26], thus indicating that IF testing and a two-step approach are not needed.

## Novel Findings in Genetics and Pathomechanisms

The pathogenesis of AAV is multifactorial and incompletely understood; however, a genetic component undoubtedly exists. There is compelling evidence that patients with MPO- and PR3-ANCA serotype exhibit different genetic association [27]. Published in 2012, the first genome-wide association study (GWAS) has provided robust evidence that GPA and MPA are different diseases and that the ANCA serotype more strongly correlates with genetic associations compared to the disease entities, further fueling the concept of a serology-based classification [28].

Further, the role of complement in the pathogenesis of AAV has only recently received attention. In a study including 98 patients (45 MPO-ANCA positive, 53 PR3-ANCA positive), Wu et al. [29••] studied activation of the alternative pathway in detail. Main differences in complement activation profile correlated predominantly with disease activity and barely with ANCA serotype. A recent study including a meta-analysis indicated that circulating complement activation products are elevated in both diseases, GPA and MPA, independent of clinical phenotype or ANCA serotype, indicating that complement activation is crucially involved in the pathogenesis of both diseases [30].

Monitoring the inflammatory status of the diseases using C-reactive protein (CRP) and erythrocyte sedimentation rate (ESR) is helpful. Both parameters tend to be higher among patients with PR3- as compared to patients with MPO-ANCA [31]. Using data from the RAVE trial, Berti et al. [32••] reported distinct cytokine profiles in patients with PR3- and MPO-ANCA positivity. Specifically, of the 186 cases included in the study, 124 exhibited PR3-ANCA, and the remainder MPO-ANCA. In this cohort of patients with active disease, immune mediators were more strongly associated with ANCA specificity than with clinical features, again underpinning the importance of ANCA in classifying these diseases.

## Differences in Demographic Factors and Manifestation

ANCA status is also a major determinant of the clinical presentation. Although all age groups can be affected, PR3-ANCA positive patients are on average 10 years younger than patients with MPO-ANCA; likewise, PR3-ANCA is significantly associated with male gender [33, 34]. Ethnicity has a role in disease manifestation, as illustrated by the predominance of MPO-ANCA among patients with either GPA or MPA in Asia.

More than 20 years ago, Franssen et al. [13] reported a higher number of extrarenal organ involvement in PR3-ANCA positive patients compared with their MPO-positive counterparts (mean number of affected organs at presentation 3.9 vs. 2.2, respectively) leading to a higher initial BVAS score in the former as compared to the latter. A large study involving 502 patients with AAV confirmed these findings [35]. These authors were also among the first to recommend that ANCA serotype should be used in conjunction with the terms MPA, GPA, and pauci-immune glomerulonephritis (GN), in categorizing patients within the AAV spectrum [35].

Ear, nose, and throat, as well as eye involvement, are more common in GPA and PR3-ANCA positive patients [31]. A recent cross-sectional study using data from the Diagnostic and Classification Criteria in Vasculitis (DCVAS) study underlined the importance of skin lesions, which occur in a third of patients with AAV at presentation. Cutaneous manifestations correlate with disease severity in patients with PR3-ANCA but not in MPO-ANCA positive subjects. In the former group, petechiae/purpura were the most frequent findings (18.6%), while the latter more frequently exhibited livedo reticularis and livedo racemosa. This may represent a useful distinguishing feature [36].

As the predominantly affected organ systems, specific differences of kidney and lung involvement will be discussed more extensively in the following sections.

## Kidney Involvement

Renal vasculitis, i.e., pauci-immune rapidly progressive crescentic GN, is a common manifestation of AAV, affecting > 75% of patients in the early course of the disease [37, 38]. Seen from the other side, AAV-GN accounts for the majority (e.g., 80% in a contemporary Korean cohort [34]) of cases of crescentic GN. It carries an unfavorable prognosis, as it confers increased risks of end-stage kidney disease (ESKD) and mortality [15, 39, 40]. ANCA are nearly always present in AAV-GN, and a titer rise better predicts relapse in patients with than in patients without renal disease [2].

Overall, prevalence of kidney involvement appears to differ between PR3- and MPO-positive patients; moreover, important clinical differences exist between patients with these two serotypes. As found in recent literature, MPO-ANCA positive patients frequently have more advanced kidney disease at presentation and show a lesser likelihood of renal recovery following treatment. A recent publication from Weiner et al. [34] reported higher glomerular filtration rate at diagnosis in PR3-ANCA positive patients. Another study, conducted by the EUVAS group, analyzed 173 kidney biopsies and reported more pronounced glomerulosclerosis, interstitial fibrosis (IF), and tubular atrophy (TA) in individuals with MPO- than in those with PR3-ANCA. In contrast, the proportion of fibrinoid necrosis or crescents was similar in both groups [41, 42]. Therefore, in AAV, kidney biopsy is not only of value for diagnostic purposes but may also provide important prognostic information at baseline.

The histopathologic classification promoted in 2010 by a panel of kidney pathologists (Berden classification) distinguishes four categories of glomerular lesions: focal (> 50% normal glomeruli), crescentic (> 50% cellular crescents), mixed (any other combination), and sclerotic (> 50% sclerotic glomeruli). Patients in the focal class tend to experience the best, while patients in the sclerotic class the worst renal outcomes [43]. It is important to note that neither data pertaining to ANCA serotype nor IF/TA or kidney function included in the score are available from this key publication, all of which have subsequently been shown to hold prognostic value too. In patients classified as sclerotic, entry GFR and TA seemed to correlate best with kidney function at 12 months and renal survival [44]. Long-term kidney survival, defined as outcome after 10 years, of the crescentic (86%) and mixed class (80%) is similar according to a recent publication. Kidney survival of the focal class was better, while the rate was lower in the sclerotic class [45•].

Several validation studies and a meta-analysis have been published since, predominantly confirming and expanding the findings described above [46]. Dividing patients by ANCA type, a large single-center study found the MPO-ANCA serotype was more frequent in the sclerotic class; furthermore, kidney survival was reduced in these subjects [47]. A subsequent publication confirmed these results in a larger, binational cohort of 136 patients [33]. Again, MPO-ANCA serotype was associated with more severe renal disease when compared with PR3-ANCA. Keeping with a more chronic or smoldering course, MPO-ANCA positivity was also associated with higher IF/TA scores, and more biopsies belonged to the sclerotic or mixed class [38]. At follow-up, ESKD and CKD stage 4 developed more frequently at 1 and 2 years in MPO-ANCA positive patients, compared to PR3-ANCA positive subjects. Further, a recent international study investigated predictors of renal outcomes in the sclerotic class, AAV-GN. Of the 50 patients included, 41 (82%) were positive for MPO-ANCA; kidney impairment was severe in this cohort with a median eGFR at entry of 14.5 (IQR 9–19) ml/min/1.73 m^2^ [44].

PR3-ANCA positive patients more often exhibit active inflammatory lesions yielding a better response to therapy [18, 41]. Conversely, in MPO-AAV, worse kidney function implies smaller risk of relapse. This has implications for the optimal duration of maintenance therapy as these patients might not benefit from long-term immunosuppression. Recently, Brix et al. [48] proposed an “ANCA renal risk score” based on 3 clinicopathologic features, namely percentage of normal glomeruli, the amount of IF/TA, and baseline eGFR. Although the prevalence of ANCA serotype was balanced among study subjects, further analyses in larger cohorts are necessary to confirm that this score is applicable to both ANCA subtypes.

Renal limited vasculitis (RLV), i.e., isolated pauci-immune crescentic GN, is a distinct subset of AAV, in which MPO-ANCA positivity is found in 80%, 10% are PR3-ANCA positive, with the remainder seronegative [49]. Interestingly, a recent study using mass spectrometry found a more pronounced complement deposition in kidney biopsies of patients with ANCA-negative GN in comparison to PR3-ANCA or MPO-ANCA positive patients with GN. The authors hypothesize that ANCA-negative GN may be triggered or promoted by a defect in the alternative complement pathway, either genetically or acquired [50]. These findings await replication in prospective studies.

A recent study investigated cases with “slowly progressive” renal vasculitis (defined by a < 50% decrease of eGFR over a 6-month period prior diagnosis). These patients were frequently MPO-ANCA positive (37/39) and involvement of other organs was absent in most patients. Most kidney biopsies were either classified as sclerotic or mixed class. Immunosuppression led to an improvement of eGFR of > 25% from diagnosis in 59% of patients [51].

In summary, kidney response to therapy appears to be better in PR3-ANCA positive patients and translates into a more favorable long-term prognosis. The renal course of MPO-AAV is characterized by a more chronic process, with global sclerosis and advanced fibrosis on histology bearing witness of a gradual accrual of damage. Therefore, the component of chronicity, rather than the degree of disease activity, provides better information regarding renal outcome [52].

A kidney biopsy exhibits prognostic value, while in cases with clinical presentations that are compatible with AAV, a positivity of either PR3- or MPO-ANCA and a low suspicion for secondary vasculitis forms, treatment can be initiated without a biopsy-confirmed diagnosis [53]. This is of particular interest in scenarios when centers aim to avoid biopsy (i.e., morbid obesity or presence of a single kidney) (Table 1).

**Table 1:** Characteristics of kidney involvement in ANCA-associated vasculitis

Characteristics of kidney involvement in AAV	MPO-ANCA	PR3-ANCA
Baseline eGFR (at diagnosis)	Lower	Higher
Histopathology	Advanced damage; higher IF/TA scores; more frequently “sclerotic” class (according to the Berden classification)	Active inflammatory lesions; more frequently “focal” class (according to the Berden classification)
Renal limited vasculitis (proportion of patients)	80%	10%
Treatment response (kidney function recovery)	Worse	Better
Long-term prognosis	Worse, chronic process	Better
Relapse risk	Lower	Higher

Abbreviations: *AAV*, ANCA-associated vasculitis; *MPO*, myeloperoxidase; *PR3*, proteinase 3; *eGFR*, estimated glomerular filtration rate; *IF/TA*, interstitial fibrosis/tubular atrophy

## Pulmonary Involvement

Lung involvement appears prominently among the various manifestations of AAV. At the most severe end of the disease spectrum, diffuse alveolar hemorrhage (DAH), as a consequence of capillaritis, carries a high mortality. Notably, the likelihood of DAH did not segregate according to ANCA specificity in a single-center cohort study of 73 patients [54]. Less dramatic pulmonary pathology ranges from segmental bronchial wall thickening, bronchiectasis, and single parenchymal nodules to multiple large pulmonary granulomas (primarily in patients with GPA) [55•].

An association of AAV with interstitial lung disease (ILD) was initially noted in 1990, and this observation was subsequently bolstered by various case series from Japan and other countries [56, 57]. A single-center study involving 53 patients with ILD who had ANCA measured found 19 ANCA positive patients, 17 of them exhibiting the MPO-ANCA subtype [58]. In a multicenter retrospective study, Comarmond et al. [59] described 49 patients with AAV and ILD. Male predominance was noted (30 men [61%], and 28 subjects exhibited kidney involvement [57%]). Again, ANCA specificity was mostly MPO (43 [88%]), with only 3 patients showing PR3-ANCA. Schirmer and colleagues [60] identified 144 patients with MPA from their Bad Bramstedt cohort, of which 12% demonstrated ILD at disease onset and 15% during long-term follow-up. It has been repeatedly stressed that the evidence of ILD often predates the manifestation of systemic vasculitis. Specifically, patients with seemingly “idiopathic” pulmonary fibrosis may present with rapidly progressive GN years after the initial diagnosis of lung disease [61]. Seroconversion, i.e., the development of ANCA in previously autoantibody negative patients with ILD, is well recognized. In a cohort of 61 patients with idiopathic pulmonary fibrosis, 6 individuals became MPO-ANCA positive during follow-up, with a median duration of 23 months (range, 0 to 71 months); 2 patients developed crescentic GN and subsequently received a diagnosis of MPA [62].

Usual interstitial pneumonia (UIP) is the predominant radiologic pattern of MPA-ILD: the FVSG reported 62 patients with AAV-ILD (89% MPO-ANCA subtype). ILD was defined as UIP (63%) or non-specific interstitial pneumonia (NSIP) according to radiologic pattern [63]. In this cohort, UIP conferred a poor prognosis.

Individuals with MPA-ILD are usually older than patients with MPA without ILD and pure “vasculitic” presentation, i.e., acute onset of constitutional symptoms. Extant literature, albeit limited, consistently indicates a slight male preponderance.

Subclinical alveolar hemorrhage has been implicated as the mechanism leading to pulmonary fibrosis. In addition, MPO-ANCA per se may play a role in the pathogenesis of pulmonary fibrosis [64]. According to another hypothesis, the chronic inflammatory process of pulmonary fibrosis may result in neutrophil destruction and subsequent development of ANCA. Tobacco exposure appears to be an additional trigger, as a high prevalence of AAV is consistently found in former or current smokers, especially among MPO-ANCA positive individuals. In contrast, no such association was found in patients with PR3-ANCA positive AAV [65••].

There is no specific treatment for AAV-associated ILD, and available literature is conflicting concerning the possible benefit of immunosuppressive therapy [64]. Nintedanib, a tyrosine kinase inhibitor, is an interesting agent in this indication given its efficacy in scleroderma lung disease [66].

PR3-ANCA positive patients with lung involvement exhibit a substantially increased risk of relapse when compared to subjects with MPO-ANCA; it is speculated that this risk can be partly reduced by trimethoprim/sulfamethoxazole, presumably by preventing respiratory tract infections [67].

To conclude, pulmonary fibrosis can be associated with MPO-AAV, and it appears to be a distinct manifestation causing increased disability and poor prognosis. Greater awareness of this association and more liberal testing for ANCA in ILD may result in an earlier recognition and, ultimately, improved outcomes. Close collaboration between pulmologists, rheumatologists, and nephrologists is clearly necessary.

## Treatment Response and Risk of Relapse

The treatment armamentarium has increased during the past years, and a particular focus was to reduce the risk of relapse once remission has been obtained by induction therapy. A study by Mahr et al. summarized findings from different clinical trials conducted by EUVAS and FVSG and indicated a higher risk of disease relapse in patients with PR3-ANCA than in other AAV patients [68]. Continuous improvements in treatment strategies have transformed AAV from an almost inevitably fatal disease to an entity with a relapsing and remitting course; concomitantly, treatment-related toxicity has emerged as a major concern. Following the publication of RITUXVAS and RAVE, two key trials paving the way for authorization of rituximab for the treatment of AAV [6, 7], significant differences in the response to treatment in AAV have become apparent. While the RAVE trial found non-inferiority of rituximab (RTX) in the induction of remission, compared to a cyclophosphamide (CYC)-based regime, the presence of PR3-ANCA (as compared to MPO-ANCA) portended a better response to RTX than to CYC [6]. This came at the cost of an increased relapse risk, necessitating prolonged maintenance therapy. Major guidelines now recommend a longer course of maintenance therapy in patients with PR3-ANCA and advocate that RTX should be given preference over CYC in induction of such cases [69, 70]. In MAINRITSAN3, a trial assessing the benefit of prolonged maintenance therapy with RTX, relapses were common in patients with PR3-ANCA vasculitis in the placebo group, underscoring the importance of long-term treatment with rituximab in these patients to prevent disease recurrences [71•]. In addition, there may be differences in response to mycophenolate mofetil (MMF) in MPO-ANCA compared to PR3-ANCA patients. The MYCYC trial (comparing MMF versus CYC for induction of remission in AAV) demonstrated non-inferiority of MMF in comparison to CYC. The study included 140 patients, with MPO-ANCA positive subjects evenly split between the two treatment arms (27/70 MMF group, 26/70 CYC group). A post-hoc analysis found no difference between MMF and CYC in the relapse rate of MPO-positive cases, while relapse risk in the PR3-ANCA vasculitis patients tended to be higher in the MMF arm [72].

Studies assessing novel steroid-sparing strategies are underway with avacopan, an antagonist of human C5aR/CD88 being one of the most promising drugs. It has shown promising results in a phase 2 trial (CLEAR), showing comparable efficacy by replacing high dose glucocorticoids [73••]. Numerically, the response rate was greater in subjects with MPO-ANCA vasculitis. The results of a large phase 3 trial (ADVOCATE) are awaited. Lastly, the PEXIVAS trial [5••] did not report any effect of ANCA type on the response to plasma exchange or on the two different corticosteroid arms.

Overall, predictors of relapse such as ANCA type have influenced treatment algorithms and should allow for a more individualized long-term therapeutic approach.

## Cardiovascular Disease and Venous Thromboembolism

With inflammation of blood vessels as their histological hallmark feature, it is not surprising that AAV confer an increased risk of cardiovascular (CV) morbidity and mortality [74]. The rate of CV events (~20% within 40 months following diagnosis) even exceeds the number observed in patients with CKD, a cohort prone to CV morbidity [75]. A recent publication, including 484 patients with a new diagnosis of AAV between 2002 and 2017, found a greater risk of death due to CV disease in MPO-ANCA+ patients compared with PR3-ANCA+ patients. The association persisted after adjustment for baseline kidney function, age, and sex [76••]. This paper corroborated an earlier publication from the EUVAS, as well as the FVSG, which highlighted (1) the increased risk of death of patients with CV involvement and (2) that the risk of death is higher in patients without PR3-ANCA [68]. A recent post-hoc analysis of the RAVE trial investigated changes in the lipid profile of patients from baseline to month 6. In patients with newly diagnosed AAV, there was a significant increment in low-density lipoprotein (LDL)-cholesterol following remission induction, and these changes were most evident in patients with PR3-ANCA vasculitis [77]. This finding implies that the individual CV disease risk should be assessed once remission is achieved. In addition, a German cross-sectional multicenter study focused on the management of CV risk factors in patients with AAV. By using guidelines issued by several authorities to treat hyperlipidemia (KDIGO and ESC), only a minority (< 25%) of the 53 patients reached their individual target LDL-cholesterol [78]. More recently, target LDL-cholesterol levels and blood pressure for patients at high risk for CV events have been lowered. More studies in the field of AAV are needed to provide evidence that a more stringent control of CV disease risk factors can prevent events.

A hypercoagulable state of patients with AAV was reported during phases of active disease and remission. The frequency of venous thromboembolic events (VTE) is up to 17.3% (Table 2), as reported in a recent study focusing on patients with ANCA-associated GN. The median time between diagnosis of vasculitis and VTE was 3 months. In this study, the absence of statin therapy was associated with an increased risk of VTE, a finding which needs to be confirmed by additional research [87]. Post-hoc analysis of the RAVE trial indicated that 16 of 197 patients had at least one event and that most VTE occurred early after enrolment (with an overall average time to event of 1.5 months). A strong association of VTE with PR3-ANCA positivity, pulmonary hemorrhage, and urinary red blood cell casts was reported [86•]. In contrast, Stassen et al. found an association of VTE with MPO-ANCA positivity and a diagnosis of MPA. No adjustment for biases such as older age of the participants was made for this finding [81]. A potential mechanism which predisposes patients with PR3-ANCA vasculitis to VTE was published by Bautz et al., showing that patients with PR3-ANCA positivity have higher anti-plasminogen antibody levels compared to MPO-positive patients. Moreover, five of nine patients with VTE in the PR3-ANCA group exhibited anti-plasminogen antibodies; in contrast, these were absent in patients with MPO-ANCA vasculitis and thrombosis [88]. Patients who achieved remission had impaired levels of endogenous thrombin potential, factor VIII, and tissue factor pathway inhibitor, which indicate the hypercoagulable state persists during remission [89]. While overall control of CV risk factors by lipid-lowering agents such as statins might be considered, we are not recommending initiation of statins to prevent VTE. Evidence from the general population stems from observational studies and should form the basis for studying their effects in properly designed interventional trials [90].

**Table 2:** Summary of systematically retrieved studies investigating the occurrence of venous thromboembolic events in AAV

	Merkel [79]	Allenbach [80]	Stassen [81]	Novikov [82]	Kronbichler [83]	Kang [84]	Berti [85]	Kronbichler [86•]	Isaacs*^9^	Henry [87]
Participants	180	613*^2^	198	288	417	204	51	197	162	133
Observational period (months)	18	58.4*^3^	73	-	60	70	84	-	57	-
Age	50.0	52.5*^3^	60 and 55*^4^	51 and 49*^5^	57	55	61.1*^8^	52	54.2 and 55.1*^4^	65.1
Sex	Men (60%), women (40%)	Men (56.1%), women (43.9%)*^3^	Men (59.6%), women (40.4%)	55% and 66.7% male*^6^	Men (54.4%), women (45.6%)	Men (46%), women (54%)	Men (52%), women (48%)	Men (50.8%), women (49.2%)	Men (39.5%), women (60.5%)	Men (63.2%), women (36.8%)
Disease	GPA	GPA (377), MPA (236)	GPA (143), MPA (34), RLV (21)	GPA (243), MPA (45)	GPA (231), MPA (186)	GPA (111), MPA (38), RLV (27)*^7^	GPA (23), MPA (28)	GPA (147), MPA (50)	GPA (95), MPA (56)	GPA (45), MPA (88)
ANCA serotype	Positive (80.6%)*	Positive (84.2%)*	PR3 (146), MPO (45), other (4), negative (3)	-	PR3 (236), MPO (181)	PR3 (110), MPO (77), negative (17)	PR3 (17), MPO (34), negative (5)	PR3 (131), MPO (66)	PR3 (86), MPO (71), negative (5)	PR3 (39), MPO (86), negative (8)
Frequency of VTE	16/180 (8.9%) during study*^1^	30/377 GPA (8.0%), 18/236 MPA (7.6%)	10/143 GPA (7.0%), 7/34 MPA (20.6%), 6/21 RLV (28.6%) *11/146 PR3 (7.5%), 11/45 MPO (24.4%), other (25%), negative (0%)*	20/243 GPA (8.2%), 3/45 MPA (6.7%)	20/231 GPA (8.7%), 21/186 MPA (11.3%) *26/236 PR3 (11.0%), 15/181 MPO (8.3%)*	7/111 GPA (6.3%), 3/38 MPA (7.9%), 1/27 RLV (3.7%) *9/110 PR3 (8.2%)*	6*^8^ (10.3%)	14/147 GPA (9.5%), 2/50 MPA (4%) *15/131 PR3 (11.5%), 1/66 MPO (1.5%)*	16/95 GPA (16.8%), 6/56 MPA (10.7%) *18/86 PR3 (20.9%), 4/71 MPO 5.6%)*	8/45 GPA (17.8%), 15/88 MPA 17.0%) *3/39 PR3 (7.7%), 18/86 MPO (20.9%)*
Time to venous thromboembolic event	2.07 months	5.8 months	8.8 months	-	-	-	-	1.5 months	1 month	3 months
Risk factors (univariate)	Older age (57.5 versus 48.6 years)	Older age (58.2 versus 52.1 years); male sex, history of VTE, stroke with motor deficit, nephrotic range proteinuria, **lower limb motor neuropathy**	**GPA (diagnosis) and PR3-ANCA positive (negative)**	-	Baseline creatinine, BVAS, cutaneous, gastrointestinal, mucous membrane/eyes, -subsequent cancer	-		PR3-ANCA positive, heart involvement, pulmonary hemorrhage, red blood cell casts	Length of follow-up, PR3-ANCA, weight, BMI, obesity, rapidly progressing GN, proteinuria, hypoalbuminemia, BVAS	Serum albumin, **statin therapy**
Risk factors (multivariate)		Older age; male sex, history of VTE, stroke with motor deficit, **lower limb motor neuropathy**		-	Baseline creatinine, CRP, cutaneous, gastrointestinal	-	-	PR3-ANCA positive, heart involvement, pulmonary hemorrhage, red blood cell casts	BMI, PR3-ANCA, hypoalbuminemia	**Statin therapy**

*Specificity not reported; *^1^ another 13 patients had a history of VTE before enrolment; *^2^ this study included 232 patients with EGPA and 285 patients with polyarteritis nodosa; *^3^ analysis is given for all patients (1130); *^4^ age was given for patients with VTE and those without; *^5^ age for the group of patients with GPA and MPA; *^6^ sex is given for patients with VTE only; *^7^ patients with EGPA, 9 double-positive, defined as patients with AAV and anti-GBM antibodies, and 5 with overlap syndrome were included; *^8^ patients with EGPA were included; *^9^ Isaacs B, et al. Kidney 360 April 2020, 1(4):258-262; DOI: 10.34067/KID.0000572019. The respective ANCA serotype is in italics. Negative associations are in bold

In summary, as treatment approaches allow for control of the disease in most patients, a particular focus is on comorbidities and complications of the diseases. CV mortality is high and a better control of risk factors seems warranted. A specific approach to reduce the burden of thromboembolic events needs to be developed, and clinical trials should be initiated to reduce the occurrence of VTE in AAV patients.

## Summary

The past years have witnessed impressive advances in the understanding of AAV, and from this, considerable improvements in patient care have followed. In parallel, a shift in classification has occurred, moving away from phenotype (GPA or MPA) and focusing instead on ANCA serotype. There is now largely consensus that PR3-AAV and MPO-AAV represent separate entities in many practical regards and differ genetically. Currently, there are no validated diagnostic criteria for AAV, likewise, an ideal classification of this entity remains elusive and relevant questions remain unsolved. For instance, the pattern of organ involvement does not always follow ANCA serology clearly. Moreover, up to a third of patients with features consistent with AAV are ANCA-negative by routine tests, and how to best treat and follow-up such patients currently remains unclear. The ongoing DCVAS study (NCT01066208) [91], a multinational, observational study to develop diagnostic and update classification criteria in vasculitis, is expected to settle some of these issues.

As is true for all diseases whose spectrum is broad, caution is required in all efforts to ascribe patients to simple disease subsets. New insights into pathophysiology, such as the role of complement in AAV, might impact future categorization. Such influence would emulate the discovery of ANCA almost 40 years ago.

The general classification of the vasculitides, and of the AAV in particular, will continue to evolve. Better insight into individual patient risk factors for disease, response to therapy, and risk of adverse events may result from this. Currently, widespread uptake of an ANCA-based classification of the AAV could rapidly usher an era of personalized medicine in this field. Such patient-tailored therapy could enable a more individualized approach with better treatment response and less therapy-related side effects ultimately resulting in improved patient-related outcomes.
